# Operational costs of four-dimensional flow cardiovascular magnetic resonance: A break-even analysis

**DOI:** 10.1016/j.jocmr.2025.101928

**Published:** 2025-06-25

**Authors:** Joshua Engel, Tyler A. Jacobson, Michael Markl, Bradley D. Allen, Maria Ibanez

**Affiliations:** a Department of Radiology, Feinberg School of Medicine, Northwestern University, Chicago, Illinois, 60611, USA; b Kellogg School of Management, Northwestern University, Evanston, Illinois, 60208, USA; c Department of Medicine, Medical College of Wisconsin, Milwaukee, Wisconsin, 53226, USA; d Department of Biomedical Engineering, McCormick School of Engineering, Evanston, Illinois, 60208, USA

**Keywords:** Financial analysis, Break-even, Reimbursement, 4D Flow CMR

## Abstract

**Background:**

Aortic hemodynamics derived from four-dimensional (4D) flow cardiovascular magnetic resonance (4DF) have shown advantages for risk stratification and treatment planning. However, clinical adoption has been limited due to high costs, long scan and post-processing times, and low reimbursement. We quantify these barriers to identify break-even points and assess methods of improving clinical adoption.

**Methods:**

We modeled the costs, resource utilization, and reimbursement under case scenarios of base cardiovascular magnetic resonance (CMR) scans, magnetic resonance angiography (MRA), adult congenital, and 4DF add-on to the CMR protocol. Price estimates were generated from market research by the purchasing department at an academic medical center. Time estimates were derived from workflow efficiency studies at the same center. Reimbursement rates were from the Centers for Medicare and Medicaid Services for 2024b. The opportunity cost of 4DF was calculated as the margin from utilizing incremental scanner time for a mixture of alternate studies. Break-even points were modeled as the number of scans needed for the yearly margin of performing 4DF to exceed performing the base scans alone. Sensitivity analyses were performed for ranges of current procedural terminology (CPT) 75565 reimbursement code, 4DF exam time, and 4DF annual software cost.

**Results:**

The incremental variable cost per 4DF scan was $101.40 at a scan time of 15 min, after accounting for the opportunity cost of scanner utilization. Break-even points were calculated across reimbursement ranging from $40–65/unit of CPT 75565, annual software costs of $10,000–$70,000, and 4DF scan times of 5–20 min. At a scan time of 15 min and reimbursement of $47.87/unit of CPT 75565, break-even points ranged from 112–778 scans as software cost increased from $10,000–$70,000. Highly accelerated 4DF with a 5-min scan time would break-even with about half of the scans, with break-even points ranging from 64–444 as software costs increased from $10,000–$70,000.

**Conclusion:**

Long scan times and high resource utilization limit the financial viability of 4DF outside of high-volume academic medical centers. Further development of accelerated 4D flow imaging techniques and expanded reimbursement criteria are needed to enable wider clinical adoption.

## Graphical abstract

**Figure:**
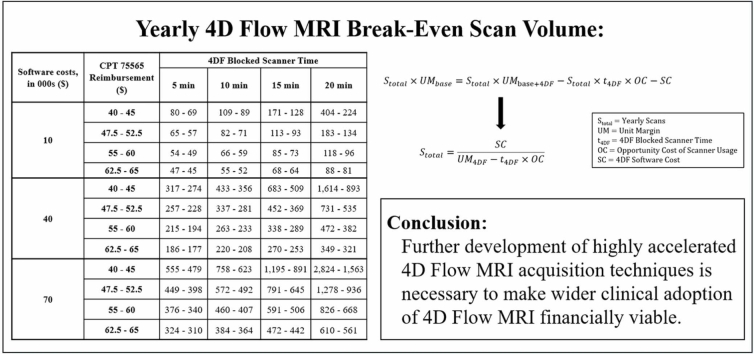

## 1 Introduction

Aortic hemodynamics derived from 4D flow cardiovascular magnetic resonance (CMR 4DF) have shown advantages for risk stratification and treatment planning for patients with thoracic aortic disease [1]. However, clinical adoption has been hindered by multiple factors, including high costs, long scan and post-processing times, and low reimbursement [2]. Clinical use has been limited to large centers that serve as tertiary referral sites for patients requiring surveillance by Multidisciplinary Aortic Teams [3]. Patients with congenital heart diseases and thoracic aortic disease require lifelong imaging surveillance per current guidelines and may benefit from the use of 4DF. As evidence grows for the predictive value of 4DF-derived biomarkers [4], the capacity for 4DF may need to extend past large academic centers. In this study, we sought to model the costs of implementing 4DF and provide yearly scan volumes necessary for 4DF to be financially viable across multiple scenarios.

## 2 Methods

The costs, resource utilization, and reimbursement under scenarios of base CMR scans magnetic resonance angiography (MRA), and adult congenital (CMR and MRA), and 4DF add-on acquisitions were modeled. Cost estimates were generated from market research by the purchasing department at an academic medical center for 2024 (Northwestern Medicine, Chicago, Illinois, USA). Scan time estimates were derived from scheduling practices at the same center [5]. Hourly wage rates were estimated using 2024 rates from the US Bureau of Labor Statistics [6], [7]. Base scans (coded as current procedural terminology (CPT) 75561 for CMR, 71555 for MRA, 75561 and 71555 for adult congenital, and 96374 for intravenous line placement) were assumed to include contrast-enhancement, with time for nursing placement of an intravenous line occurring ahead of scheduled scanner time (and excluded from scan time). Reimbursement for 4DF (CPT 75565) was taken from the 2024b Medicare fee schedule. An average hourly margin from CMR scanner use was generated from estimated utilization by study type derived from Petroianu et al. [8]. Costs for alternate scans assumed contrast-enhancement (except for non-spine orthopedic exams) and no additional software requirements. The average hourly margin was calculated as the mean of each study’s hourly margin weighted by frequency.

**Fig. 1:**
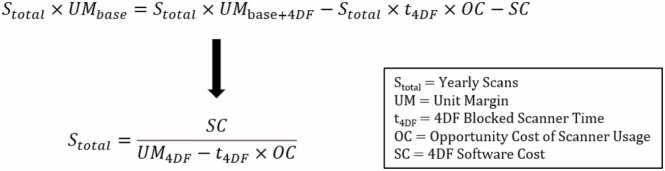
Break-even equation used to model the number of yearly 4DF add-on scans needed for the yearly margin of performing 4DF add-on to exceed performing the base scans alone. UM_base_ represents the gross profit of performing each base scan alone; UM_base_ × S_total_ represents the total yearly gross profit of performing base scans without 4DF. *4DF* 4D flow CMR, *4D* four-dimensional, *CMR* cardiovascular magnetic resonance

Break-even points were modeled as the number of yearly 4DF add-on scans needed for the margin of performing 4DF add-ons to exceed performing base scans alone. The yearly margin of 4DF add-on was calculated as the contribution margin of scan volume subtracted by the opportunity cost of incremental scanner time and incremental yearly software cost. Break-even points were calculated using the equation detailed in Fig. 1. We performed sensitivity analyses to compute break-even points under ranges of 4DF reimbursement, 4DF exam time, and incremental yearly cost of 4DF post-processing software. We also compute the initial profit margins for a theoretical adult imaging center performing 500 4DF scans/year (average volume per scanner at an academic medical center) with one post-processing software license.

## 3 Results

**Fig. 2:**
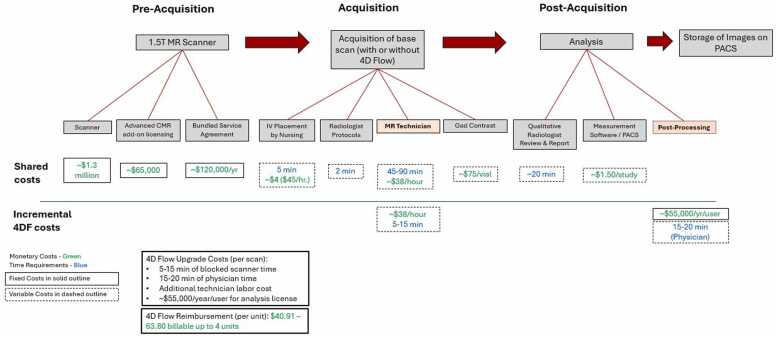
Workflow cost diagram for the shared (top row) and incremental costs (bottom row) of base CMR exams (CMR, MRA, adult congenital) and 4DF add-on scans. Monetary costs are denoted in green and time costs are denoted in blue. Fixed costs are outlined in solid line text boxes and marginal costs are outlined in dashed line text boxes. Cost figures are given as representative examples in 2024 United States dollars. *CMR* cardiovascular magnetic resonance, *MRA* magnetic resonance angiography, *4DF* 4D flow CMR, *4D* four-dimensional, *MR* magnetic resonance, *PACS* picture archiving and communication system, *CMR* cardiovacular magnetic resonance, *IV* intravenous

A flowchart representing an example of the fixed, marginal, and time costs of the base scans with 4DF add-on is shown in Fig. 2. Performing 4DF incurred marginal cost from incremental technician labor, yearly incremental software cost, and increased scanner occupancy. The opportunity cost of MRI scanner utilization was $367.65/h (Supplemental Table 1). At a 15-min 4DF scan time, the incremental variable cost per 4DF scan was $101.40 after accounting for the opportunity cost of scanner utilization. Medicare reimbursement for 4DF was modeled from $40.91–$63.80 per unit of CPT 75565.

Break-even points across ranges of reimbursement rates, 4DF scan times, and software costs are presented in Table 1. Manual calculation of break-even points with individual inputs is possible using the Excel spreadsheet provided in the Supplementary Material. Break-even volumes decreased with increasing reimbursement rates, shorter scan times, and decreasing software costs. With reimbursement set to $47.87 for each four units of CPT 75565, software cost of $55,000, and scan time of 15 min, the break-even point was 611 scans. Holding other parameters constant, increasing reimbursement to $55 resulted in a break-even point of 464 scans, shortening scan time to 5 min resulted in 349 scans, and reducing software cost to $25,000 resulted in 278 scans.

**Table 1:** Break-even 4DF yearly scan volumes across ranges of software cost, reimbursement per unit of CPT 75565, and 4DF blocked scanner times.

Software costs, in 000s ($)	CPT 75565 reimbursement ($)	4DF blocked scanner time						
		5 min	7.5 min	10 min	12.5 min	15 min	17.5 min	20 min
10	40–45	80–69	92–78	109–89	133–105	171–128	240–163	404–224
	47.5–52.5	65–57	72–63	82–71	95–80	113–93	140–110	183–134
	55–60	54–49	60–53	66–59	74–65	85–73	99–83	118–96
	62.5–65	47–45	51–48	55–52	61–57	68–64	76–71	88–81
25	40–45	199–172	229–194	271–223	332–262	427–319	600–406	1009–559
	47.5–52.5	161–142	180–157	205–176	237–200	283–231	349–273	457–335
	55–60	135–122	148–133	165–146	185–161	211–181	246–206	295–239
	62.5–65	116–111	126–120	138–130	152–143	169–158	190–177	218–201
40	40–45	317–274	366–310	433–356	530–419	683–509	960–649	1614–893
	47.5–52.5	257–228	288–252	337–281	380–319	452–369	558–437	731–535
	55–60	215–194	237–212	263–233	296–258	338–289	394–329	472–382
	62.5–65	186–177	201–192	220–208	242–228	270–253	304–283	349–321
55	40–45	436–377	548–426	596–490	729–576	939–700	1320–892	2219–1228
	47.5–52.5	353–313	395–346	450–387	522–439	621–507	768–600	1004–736
	55–60	296–267	325–291	361–320	406–354	464–397	541–452	649–525
	62.5–65	255–244	276–263	302–286	333–314	371–347	418–389	480–441
70	40–45	555–479	641–542	758–623	928–734	1195–891	1679–1135	2824– 1563
	47.5–52.5	449–398	503–440	572–492	664–558	791–645	977–764	1278–936
	55–60	376–340	414–370	460–407	517–451	591–506	689–576	826–668
	62.5–65	324–310	352–335	384–364	423–399	472–442	532–495	610–561

Data are total 4DF scans per year with ranges corresponding to reimbursement rates for given software cost and blocked scanner time. Four units of CPT 75565 were charged for all calculations

*4DF* 4D flow CMR*, 4D* four-dimensional*, CMR cardiovascular magnetic resonance*, *CPT* current procedural terminology

The calculation of gross profit margins for a theoretical imaging center is presented in Table 2. Yearly 4DF software cost was modeled as $55,000, with 15 min of 4DF scan time reimbursed at $47.87/unit of CPT 75565. At 500 yearly 4DF scans, charging 4 units of CPT 75565, performing 4DF incurred a $9970 loss after accounting for opportunity cost. Break-even was not possible at 1 unit of CPT 75565 at current reimbursement rates.

**Table 2:** Calculation of gross margins for cardiac MRI (CMR), MRA, adult congenital scans, and 4DF add-on scans for an example imaging center.

	CMR	MRA	Adult congenital	4D flow add-on	
				75565 × 1	75565 × 4
Reimbursement/scan ($)	**423.01**	**392.11**	**777.10**	**47.87**	**191.48**
-Unit marginal cost ($)	**108.73**	**108.73**	**137.21**	**9.49**	**9.49**
=Unit contribution margin ($)	314.28	283.38	639.90	38.38	181.99
Scanner block time (h)	**0.75**	**0.75**	**1.5**	**0.25**	**0.25**
UCM/h = hourly margin ($)	419.04	377.84	426.60	153.52	727.96
*Yearly scans	*500*	*500*	*500*	*500*	*500*
Yearly contribution margin ($), per MRI scanner	157,140	141,690	319,950	19,190	90,995
-Incremental SC	**157,140**	**141,690**	**319,950**	**(35,810)**	**35,995**
MRI occupancy (h)	375	375	750	125	125
MRI opportunity cost ($)	0.00	0.00	0.00	45,956.25	45,956.25
Margin—opportunity cost ($)	157,140	141,690	319,950	(81,766.25)	(9970.25)

Data values represent mean estimates. Reimbursement is set to Medicare rates in 2024b for Chicago, Illinois, 4DF scan time is 15 min, and software cost is set to $55,000. CMR and MRA scans are blocked for 45 min and adult congenital scans are blocked for 90 min. Bold values indicate inputs taken from the model. Italics values indicate set parameters

*UCM* unit contribution margin*, SC* software costs (negative)*, 4DF* 4D flow CMR*, 4D* four-dimensional*, CMR cardiovascular magnetic resonance, MRA* magnetic resonance angiography

## 4 Discussion

This study suggests that current reimbursement and cost inputs limit the financial viability of implementing 4DF outside of high-volume cardiac imaging centers. Each MR scanner designated for 4DF performed approximately 500 scans per year at our institution in 2024. Given this, the break-even volumes across much of the modeled ranges of input parameters may not be realistic for smaller prospective imaging centers. In our model, at 500 yearly 4DF scans, a prospective imaging center would be forgoing roughly $9970 in operating margin by utilizing the time spent on 4DF acquisition instead of their existing study mix.

The sensitivity analyses revealed targets for 4DF scan time and software costs that allow for attainable break-even scan volumes. 4DF scan times of 5–7.5 min brought break-even points below 500 scans per year across all but the lowest modeled reimbursements and highest projected software costs. While standard 4DF acquisitions are often blocked for 15 min, improvements in accelerated 4DF techniques [9] could reduce clinical scan times in the future. Moreover, break-even points were highly sensitive to decreases in yearly incremental software costs. Alternate payment models for post-processing software, such as introductory periods of per-study pricing as new imaging centers build patient volume, may promote 4DF adoption.

This study also shows that the recent change in coding guidelines for flow imaging, such as 4DF—whereby the Centers for Medicare and Medicaid Services raised the Medically Unlikely Edit value of CPT 75565 from 1–4 in 2023—was beneficial for the financial viability of 4DF. Within the model parameters, break-even was impossible with reimbursement for only one unit of CPT 75565. The coding change was therefore critical in supporting the transition of 4DF from the research setting to routine clinical practice.

While 4DF generates many advanced hemodynamic parameters, some of these metrics can also be captured using two-dimensional phase contrast (2D-PC) acquisitions, which can offer decreased scan times (1–3 min per plane). However, in patients who require placement of multiple planes in non-standard locations, including more complicated analyses such as aortic dissection or congenital heart disease, the single volumetric acquisition of 4DF reduces the burden on the MRI technician and may result in shorter scan times compared to 2D-PC, while allowing for generation of more advanced hemodynamic parameters and flexibility in post-hoc plane placement [10]. 2D-PC offers identical reimbursement to 4DF and is often bundled into a shared software package. Given the different scenarios in which 2D-PC or 4DF confers shorter scan time, optimizing the use of 2D-PC for standard CMR exams and 4DF for more complex exams would reduce average scan time and lower break-even scan volumes, should the software cost be bundled. Currently, 4DF is reimbursed only as an add-on code when CMR is billed but is not allowable for MRA-only exams. Given the increasing evidence of the utility of 4DF-derived hemodynamics in aortic disease risk stratification, expanding reimbursement eligibility for 4DF to MRA is important to support clinical adoption and financial viability.

Overall, this model shows that long scan times and high software costs limit the financial viability of 4DF. Prospective adopters of 4DF should ensure they will have sufficient study volumes or minimal incremental software costs. Additional work is needed to incentivize adoption in community settings, including improvements in accelerated 4DF techniques to minimize scan times, reductions in software costs, and/or higher reimbursement rates with expanded 4DF add-on criteria.

## 5 Limitations

The confidentiality of medical equipment and software sales may have skewed price estimates; a larger sample of prices would improve model robustness. Additionally, national median values were used because exact wage rates were unavailable. Ancillary medical staff were assumed to be paid hourly to derive marginal costs, which may not accurately reflect real-world compensation. Due to heterogeneity in cost structure, the cost of picture archiving and communication system was modeled as $1.50/scan across all scenarios. Radiologists were assumed to have sufficient capacity to read incremental 4DF scans, and marginal radiologist labor was not included in cost estimates due to often salary-based compensation structures. Pediatric exams were excluded due to anesthesia-related workflow differences. We limited the analysis to Medicare payments. The model could be improved by obtaining reimbursement rates for a real-world mix of public and private payors, which would likely increase reimbursement estimates and reduce break-even volumes. The opportunity cost calculation assumed MRI scanners were always fully utilized, MRI capacity was represented as a pooled sum across an undefined number of identical scanners, and there was no additional post-processing software cost for alternate studies. Finally, this analysis focuses on direct financial viability only and does not account for potential increases in patient willingness to pay from offering advanced imaging.

## Author contributions

**Joshua Engel:** Writing – review & editing, Writing – original draft, Methodology, Formal analysis, Data curation. **Bradley D. Allen:** Writing – review & editing, Resources, Conceptualization. **Maria Ibanez:** Writing – review & editing, Writing – original draft, Methodology, Conceptualization. **Tyler A. Jacobson:** Writing – review & editing, Writing – original draft, Formal analysis, Data curation. **Michael Markl:** Writing – review & editing, Conceptualization.

## Declaration of competing interests

The authors declare the following financial interests/personal relationships which may be considered as potential competing interests: Michael Markl reports a relationship with Third Coast Dynamics that includes board membership, employment, and equity or stocks. Michael Markl reports a relationship with Third Coast Dynamics that includes board membership, employment, and equity or stocks. Bradley Allen reports a relationship with Circle Cardiovascular Imaging Inc. that includes consulting or advisory. Bradley Allen reports a relationship with Siemens that includes travel reimbursement. Co-authors serving on editorial board for the Journal of Cardiovascular Magnetic Resonance, B.A, M.M. Co-authors receiving STTR grant: NIH/NHLBI: R42HL174259 “Anatomic Imaging Derived 4D Hemodynamics using Deep Learning”, B.A., M.M., M.I. The other authors declare that they have no known competing financial interests or personal relationships that could have appeared to influence the work reported in this paper.

## Data Availability

The data supporting the findings of this study are available on request from the corresponding author J.E.

## Footnotes

Appendix A Supplementary data associated with this article can be found in the online version at doi:10.1016/j.jocmr.2025.101928.

## References

[1] Bissell M.M., Raimondi F., Ait Ali L., Allen B.D., Barker A.J., Bolger A. (2023). 4D flow cardiovascular magnetic resonance consensus statement: 2023 update. J Cardiovasc Magn Reson.

[2] Gorecka M., Bissell M.M., Higgins D.M., Garg P., Plein S., Greenwood J.P. (2022). Rationale and clinical applications of 4D flow cardiovascular magnetic resonance in assessment of valvular heart disease: a comprehensive review. J Cardiovasc Magn Reson.

[3] Isselbacher E.M., Preventza O., Hamilton Black J., Augoustides J.G., Beck A.W., Bolen M.A. (2022). ACC/AHA guideline for the diagnosis and management of aortic disease: a report of the American Heart Association/American College of Cardiology Joint Committee on Clinical Practice Guidelines. Circulation.

[4] Rodríguez-Palomares J.F., Dux-Santoy L., Guala A., Galian-Gay L., Evangelista A. (2023). Mechanisms of aortic dilation in patients with bicuspid aortic valve. J Am Coll Cardiol.

[5] Oechtering T.H., Nowak A., Sieren M.M., Stroth A.M., Kirschke N., Wegner F. (2023). Repeatability and reproducibility of various 4D Flow MRI postprocessing software programs in a multi-software and multi-vendor cross-over comparison study. J Cardiovasc Magn Reson.

[6] Bureau of Labor Statistics USDoL. Occupational Outlook Handbook, Radiologic and MRI Technologists. https://www.bls.gov/ooh/healthcare/radiologic-technologists.htm2024. (accessed May 1, 2025).

[7] Bureau of Labor Statistics USDoL. Occupational Outlook Handbook, Registered Nurses. https://www.bls.gov/ooh/healthcare/registered-nurses.htm2024. (accessed May 1, 2025).

[8] Petroianu L.P.G., Li L., Mieloszyk R.J., Mastrangelo C.M., Stapleton S., Hall C. (2024). MRI log file analysis for workflow improvement. Curr Probl Diagn Radio.

[9] Pathrose A., Ma L., Berhane H., Scott M.B., Chow K., Forman C. (2021). Highly accelerated aortic 4D flow MRI using compressed sensing: performance at different acceleration factors in patients with aortic disease. Magn Reson Med.

[10] Sjöberg P., Hedström E., Fricke K., Frieberg P., Weismann C.G., Liuba P. (2023). Comparison of 2D and 4D flow MRI in neonates without general anesthesia. J Magn Reson Imaging.

