# Supplementary material for: Operational costs of four-dimensional flow cardiovascular magnetic resonance: A break-even analysis
Source: J Cardiovasc Magn Reson. 2025 Jun 25;27(2):101928. doi: 10.1016/j.jocmr.2025.101928 (PMC12673199; doi:10.1016/j.jocmr.2025.101928)
Supplement: Supplementary file 1 — Supplementary material [file mmc1.docx]

**Supplementary Table 1:** Calculation of opportunity cost of MRI utilization. Study mix rate derived from Petroianu, et al. (8) For simplicity, all orthopedic joint exams and lumbar and thoracic spinal exams were combined due to similar reimbursement, marginal cost, and scanner block time. UCM: unit contribution margin, wo: without contrast, w/wo: with and without contrast.

|  | **Orthopedic Joint (wo)** | **Pelvis (w/wo)** | **Spine (w/wo)** | **Abdomen (w/wo)** | **Liver (w/wo)** | **Brain (w/wo)** | **MRA Head (w/wo)** |
| --- | --- | --- | --- | --- | --- | --- | --- |
| **Reimbursement/Scan** ($) | 215.09 | 395.30 | 378.55 | 396.66 | 422.76 | 376.17 | 387.87 |
| **- Unit Marginal Cost** ($) | 24.00 | 118.82 | 114.25 | 122.33 | 114.69 | 113.31 | 113.45 |
| = **Unit Contribution Margin** ($) | 191.09 | 276.48 | 264.30 | 274.33 | 308.07 | 262.87 | 274.42 |
| **Scanner Block Time (hrs.)** | 0.50 | 0.85 | 0.75 | 0.93 | 0.76 | 0.73 | 0.73 |
| UCM/hours = **Hourly Margin** ($) | 382.18 | 324.70 | 352.40 | 295.14 | 405.44 | 360.58 | 374.81 |
| **Study Mix Rate** | 0.13 | 0.04 | 0.10 | 0.07 | 0.18 | 0.26 | 0.21 |
| **Weighted Alternative Margin/hour** ($) | 367.65 |  |  |  |  |  |  |
